# Optimization Algorithm of Urban Rail Transit Network Route Planning Using Deep Learning Technology

**DOI:** 10.1155/2022/2024686

**Published:** 2022-07-13

**Authors:** Yaqi Ma

**Affiliations:** School of Transportation, Soochow University, Suzhou 215131, China

## Abstract

Under the present background, optimizing the existing urban rail transit network is the focus of urban rail transit construction at present. Based on DL, this paper constructs the optimization algorithm of urban rail transit network route planning. According to the current urban layout and urban planning, build a suitable rail transit network line form; according to the function, the types of urban rail transit stations are divided, and the optimization of urban rail transit network lines is realized. In addition, according to the K short path algorithm, this paper calculates the effective path between any stations of rail transit and, according to the model, allocates the passenger flow to each path. Experimental results show that the accuracy of real-time traffic flow prediction by this algorithm can reach 94.98%, which is about 9% higher than other methods. This algorithm can effectively optimize the route planning of urban rail transit network. This verifies the effectiveness of the route planning optimization algorithm proposed in this paper. Using the algorithm in this paper for line planning can get good real time, rationality, and optimality.

## 1. Introduction

One of the most serious issues that has plagued cities all over the world, particularly large and medium-sized cities, is traffic congestion. In recent years, the scale of Chinese cities has grown, the urbanization process has accelerated, and urban economic development has progressed rapidly. The number and distance of people's trips have increased as the city has grown and their living standards have improved, and traffic flow has increased significantly [1]. The rationality of urban traffic network layout, the scientificity of line facility configuration, and the effectiveness of network line services are all factors in urban rail transit planning [2]. It is critical to the city's overall development, functional layout, land use, and urban form evolution. Transportation, as an important infrastructure for promoting the exchange of materials and information within urban agglomerations, is critical in providing channels for city internal development and interconnection [3]. Fast transportation has the potential to amplify the power of urban development and connect cities. Network route planning is a subset of overall urban planning that uses the overall urban planning as a guide and, at the same time, has some impact on the overall urban planning due to the characteristics of urban rail transit [4]. It is a comprehensive, long-term, and guiding macro planning that involves many specialties such as urban planning, traffic engineering, rail transit engineering, architectural engineering, and social economy. Furthermore, urban rail transit requires a significant financial investment and a lengthy construction period, all of which have a significant impact on the city's social and economic development, as well as land development and utilization. To do a good job on such a large system project, we need to do overall planning and reasonable arrangements, as well as scientific and reasonable rail transit planning, to lay a good foundation and set the stage for the follow-up work.

Due to the slow construction of rail transit and the lack of practical verification and summary, there have been some problems such as too simple planning content, strong planning color, and poor implementability. At the same time, due to the uncertain factors in the process of urban development, the early planning of some cities can no longer meet the needs of current urban development. Therefore, it is necessary to optimize the urban rail transit network and improve its performance and transportation service capacity. The concept of deep learning (DL) [5, 6] originates from the research of ANN (artificial neural network) [7] and is a new research direction of machine learning [8–10]. ANN is a mathematical model that imitates biological NN (neural network) for distributed parallel information processing [8]. In ANN, neurons connect and interact with each other to form NN. There are different ways of linking neurons in different NN types. The obtained deep network structure of DL contains a large number of neurons, which has more levels and depths than the simple NN structure, so it is intended to study more abstract data representations. The “learning” of DL mainly focuses on the abstract representation of data and finds the essential relationship between variables by constantly mining the internal structure of data. A good data representation can keep the information useful to the learning task, and at the same time, it can eliminate the influence of the change of factors unrelated to the learning task in the input data on the learning performance. DL has the advantages of high model accuracy and simple feature extraction, and it has excellent applications in speech recognition, natural language processing, image recognition, and other fields. In this paper, DL algorithm is applied to the optimization of urban rail transit network route planning, and its innovations are as follows:

1. Based on the idea that urban rail transit plays a key role in urban traffic, this paper studies the line planning theory of urban rail transit network; based on the analysis of existing practice and research results at home and abroad, and combining theory with practice, this paper puts forward solutions to several common problems in the route planning of urban rail transit network. Based on this, DL theory is used to study the optimization of urban rail transit network, which provides some reference for the optimization of urban rail transit network.
2. The disequilibrium coefficient of passenger flow distribution in the section is calculated. This paper analyzes the influencing factors of cross section passenger flow from the aspects of network line conditions, land use, passenger factors, and environmental conditions. Based on the calculated cross section passenger flow data, several cyclic DL prediction models with time series as input are established and the parameters are optimized. The verification results show that the algorithm has high prediction accuracy and high running efficiency and meets the requirements of accuracy and timeliness. It provides a brand-new perspective for the route planning algorithm of urban rail transit network.

The main structure of this article is as follows: the first chapter is the introduction. This chapter mainly expounds the research object, background, purpose, and significance of the paper, and summarizes the research methods and the structure of the paper. The second chapter introduces the related research literature of urban rail transit network route planning at home and abroad, and further leads to the research work and research methods of this paper. The third chapter is divided into two parts.  Section 3.1 compares and analyzes the development status of rail transit and introduces the related contents of DL.  Section 3.2 mainly discusses the optimization algorithm of urban rail transit network route planning based on DL. The fourth chapter is the experimental part. In this chapter, the optimization algorithm of urban rail transit network planning based on DL proposed in this paper is tested and compared with other algorithms. The performance of this algorithm is comprehensively analyzed from the experimental results. The fifth chapter is the summary and outlook. Firstly, it reviews and summarizes the research content of this paper, points out the deficiency and improvement space of the research content, and looks forward to the follow-up research.

## 2. Related Work

The construction of urban rail system is the basic facilities of a city. It is also a systematic project with large investment and long construction period. At the same time, urban rail transit, as an important part of urban public transport, has a very important impact on urban social economy and land development. Therefore, according to the future development of the city, how to plan a reasonable rail transit network line form, scale, and surrounding land development form has attracted more and more attention from government departments, research institutions, experts, and scholars.

Wang et al. calculated the line density of the rail transit network based on the population density of a city, judged the rationality of the construction scale of its network lines, classified the urban space and the layout of the rail transit network lines, and analyzed the corresponding relationship between the two [11]. Shang et al. studied the vulnerability of urban rail transit systems and the optimization of network resilience [12]. Based on the analysis of the reasonable scale of network lines and its influencing factors, Li et al. took the traffic demand and the service level of network lines as the main influencing factors of the reasonable scale of urban rail transit and carried out a reasonable scale calculation [13]. Ding et al. constructed the probability distribution function of subway passenger flow and analyzed a certain urban rail transit system as an example, showing that the station passenger flow is closely related to the development degree of the surrounding land, industry, commerce, and residence [14]. Xin et al. took the algebraic connectivity as the objective function and the construction cost as the constraint condition, established an optimization model of the urban rail transit network invulnerability, and used the improved particle swarm optimization algorithm to solve the model [15]. Based on the directed connection graph of factors affecting the scale of network lines, Hu et al. obtained the hierarchical structure of factors affecting network lines by establishing an accessibility matrix; the matrix is based on the hierarchical division of influencing factors, and the hierarchical structure of each influencing factor is obtained [16]. Li et al. believed that the overall urban passenger demand is jointly undertaken by the road traffic passenger system and the rail transit passenger system; based on this supply-demand balance principle, a reasonable scale of network lines was calculated [17]. Through in-depth research on the demand relationship of rail transit, Nie et al. came to the conclusion that rail transit demand is concentrated in urban passenger flow corridors [18]. Ding et al. took urban rail transit network construction as the research object and established an urban rail transit network optimization model [19]. The model takes the rationality and accessibility of rail transit lines as constraints and selects the minimum total travel time, the minimum total line length, and the minimum total number of transfers to establish a multiobjective function. Wang et al. believed that urban rail transit planning can be divided into three interrelated problems [20]. The first category is to study the design of rail transit network; the second category is to analyze and forecast rail transit demand; the third category is to evaluate and decide on urban transportation planning. Jeongwoo et al. studied the robustness optimization problem of urban rail transit network under uncertain demand [21]. The research proposes the concept of robustness of rail transit network under uncertain demand and establishes predictable and unpredictable models of uncertain demand.

This paper proposes an optimization algorithm for urban rail transit network route planning based on DL, and analyzes and discusses the design and implementation of the algorithm in detail, based on a thorough review of previous literature. The passenger flow is allocated to each train on each line based on the passengers' arrival and departure times, the cross section passenger flow in the statistical period is calculated, and the stability of its time series is examined in this paper. The historical data information of the corresponding date type is selected as the network's training data for different forecast periods, and the trained LSTM (long short-term memory neural network) model is used to make short-term forecasts of cross section passenger flow, with the forecasting accuracy calculated by each error index to test the model's validity. Experiments show that this algorithm is more feasible and effective than other algorithms, and that it can produce better predictions.

## 3. Methodology

### 3.1. DL and Urban Rail Transit Network Route Planning

**Figure 1:**
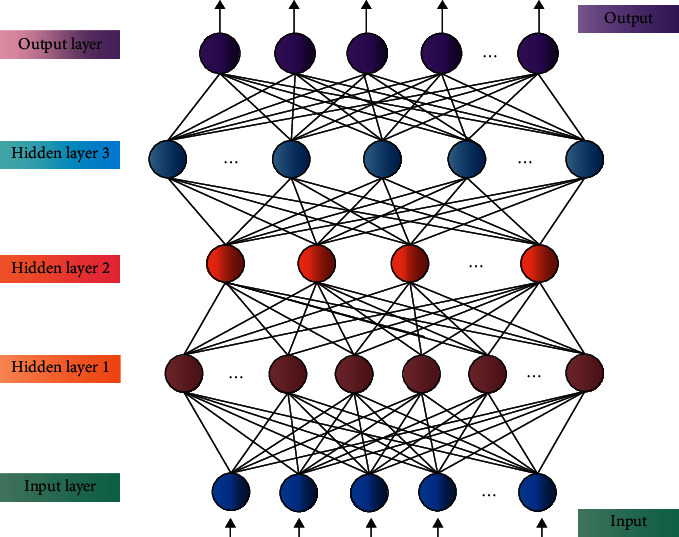
NN model.

The concept of DL comes from the study of ANN, and it is a new research direction of machine learning. ANN evolved from biological NN, and a neuron is a nerve cell. Artificial neuron is the most basic unit to process information by using NN. Theoretically, the more parameters a model has, the higher its complexity and capacity, and correspondingly it can complete more complex learning tasks. However, in general, complex models often fall into low training efficiency and are easily over-fitted, so it is difficult to be favored by researchers. Compared with the traditional NN or machine learning, DL is a relatively novel research method and research direction. The advantages of DL are high model accuracy and simple feature extraction [22]. Practice has proved that it has good effects in computer vision, natural language processing, and future trend prediction. In this paper, the DL theory is applied to urban rail transit network route planning, and an optimization algorithm of urban rail transit network route planning based on DL is proposed. The DL model increases the number of nonlinear hidden layers and transforms the original signal layer by layer to obtain new abstract features for subsequent learning tasks. DL, as a major breakthrough in realizing machine learning and accelerating human beings to the era of artificial intelligence, is based on the research in the field of NN. The obtained deep network structure of DL contains a large number of neurons, which has more levels and depths than the simple NN structure, so it is intended to study more abstract data representations. DL is also a multilevel and multistep learning process. In order to better simulate the neuron structure of human brain, more hidden layers are added, and the learned knowledge is transferred layer by layer to achieve the purpose of information stratification and systematic learning. The basic model framework of DL includes convolutional NN, stacked automatic coder, recursive NN, and deep belief network. The NN model is shown in Figure 1.

The urban population has exploded in recent years, and a large number of migrant workers have flooded into cities. Urban traffic faces severe situations and challenges due to frequent exchanges of people and materials. Road congestion, chaotic traffic order, frequent traffic accidents, and traffic environmental pollution are all serious issues in many cities. Because of its large capacity and high speed, rail transit has gradually become the development focus of transportation infrastructure in large cities in this environment. It has been instrumental in resolving traffic congestion in cities and promoting the efficient use of urban land. To fully exploit the leading role of urban rail transit in economic development, it is critical to optimize the existing urban rail transit network and build new lines in the modern environment [23]. Urban rail transit, which includes subways, light rail, monorails, trams, new traffic, and high-speed maglev trains, is referred to as rapid rail transit. Large transportation capacity, high speed, safety, reliability, and punctuality are all common characteristics. It has the ability to travel on wheels and rails on the ground, in the air, and underground. Because of these characteristics, urban rail transit has obvious advantages as a mode of urban transportation. It is especially advanced compared to other modes of public transportation when it comes to resolving traffic congestion in large and medium-sized cities, such as heavy passenger traffic during peak hours. With the rapid development of rail transit and the continuous expansion of rail network lines in recent years, urban rail transit network operation has become a new trend. Because the urban rail transit system serves as a mode of transportation, its transportation service function must be considered. At the same time, it has the nature of a network, and optimizing it solely from one perspective is not comprehensive. The basic types of rail transit usually include subway system, light rail system, suburban railway, monorail system, new transport system, tram, and automatic passenger rapid transit system. In order to reasonably select the form of rail transit system under different objectives, the basic types of urban rail transit can be classified according to different standards.

The main objectives of urban rail transit planning include ① solving the contradiction between traffic demand and supply, ② achieving the goal of urban planning, and ③ meeting the needs of sustainable development. Rail transit can guide the development of urban structure, and its principle is to greatly improve the traffic supply and guide the high-intensity utilization of surrounding land. Urban rail transit with large capacity, high speed, and independent dedicated track has already met the conditions as the backbone transportation mode of public transportation system in big cities. The backbone system is to undertake a large proportion of urban passenger transport turnover [24]. It is generally difficult for a single rail transit line to meet this backbone requirement; this is mainly because of the limitations of its attracting range of passenger flow and route direction. Therefore, rail transit must form a network to play a key role. The research of network planning involves many specialties such as urban planning, traffic engineering, rail transit engineering, architectural engineering, and social economy. It is a comprehensive, long-term, and guiding macro planning. It emphasizes the unity of stability and flexibility. Stability is to plan that the overall layout of network lines should be stable in space, central city, and time; flexibility is the extension condition of planning. Under the condition of constantly changing urban conditions, there should be room for flexible changes in the outer urban areas in space and in the long term in time. In the planning of urban rail transit, the principles of functionality, rationality, economy, and sustainable development must be followed. There are actually three ways of urban rail transit lines: elevated, ground, and underground. They all have their advantages and disadvantages. In addition, the determination of standards such as curve and slope also needs to consider the specific conditions of the line. An important part of the route planning is the station planning and site selection, which needs to consider the station attraction, station form, station spacing, cost, and other aspects. The location of depot is also an important part of route planning. It requires the approach to the trunk line as much as possible and the necessary land conditions. Urban rail transit has a huge investment and a long construction period, which has a great influence on the social and economic development of the city, land development and utilization, etc. However, due to the uncertain factors in the process of urban development, the early planning of some cities can no longer meet the needs of current urban development. Therefore, it is necessary to optimize the urban rail transit network and improve its performance and transportation service capacity.

### 3.2. Optimization Algorithm of Urban Rail Transit Network Route Planning

**Figure 2:**
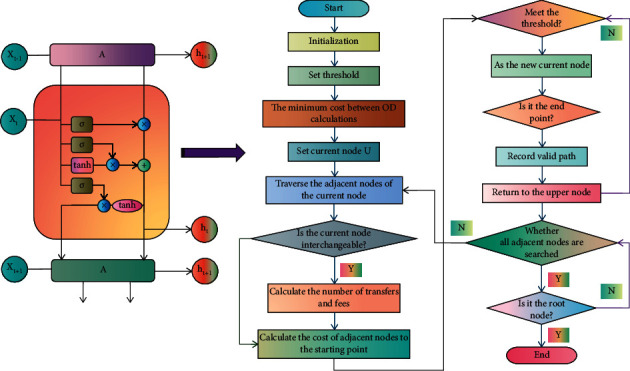
LSTM structure and algorithm flowchart.

Through DL-related content, this chapter focuses on the optimization algorithm for urban rail transit network route planning. The neuron model is the most fundamental unit in NN. According to the training data set, the NN learning process adjusts the connection weight between neurons as well as the threshold or bias of each functional neuron. NN can fit any nonlinear system, even if it only has one hidden layer. As a result, only one hidden layer is typically employed. After comparing and analyzing the fitting results of multiple hidden layers and a single hidden layer, it was discovered that the fitting results are not significantly different, so the reasonable scale of urban rail transit was calculated and analyzed using NN with three layers, including one hidden layer. However, as the prediction time step is increased, the influence of the previous time node on the current time node diminishes, and when information from a distant time node is needed in the model, the network model is unable to effectively reflect this connection; the learning ability is greatly weakened, the gradient disappears, and the model's prediction effect deteriorates. Therefore, LSTM is introduced in this paper. The LSTM structure and algorithm flowchart are shown in Figure 2.

In this paper, the SpaceL method is used to construct the urban rail transit network model. In the model, the nodes represent the urban rail transit stations and the connecting edges represent the interval routes. Transformation of rail transit network diagram into mathematical model is as follows:

(1) $$\begin{array}{c} G=\left(V,E\right). \end{array}$$

Among them, *V* represents the set of nodes. If the rail transit includes *n* stations, there are

(2) $$\begin{array}{c} \left|V\right|=\text{nV}\\ =\left(v_{1},v_{2},v_{3},\ldots ,v_{n}\right). \end{array}$$

*E* represents the set of edges. If the rail transit includes *m* section routes, there are

(3) $$\begin{array}{c} \left|E\right|=\text{mE}\\ =\left(e_{1},e_{2},e_{3},\ldots ,e_{m}\right). \end{array}$$

In a subway network, a passenger travel path consists of stations and segments, and the base travel time for a path is equal to the inbound time, ride time, outbound time, and if transfers are involved, plus the sum of the transfer times. Let *C*_*w*_^*k*^ denote the total time spent by most passengers traveling on the *k* th route between the traffic trip volume pair *W*, then

(4) $$\begin{array}{c} C_{k}^{w}=J_{o}+\sum_{i,j}T_{i,j}\delta_{ij,k}^{w}+\sum_{i}e_{i}^{l,m}\phi_{i,lm}^{w,k}+K_{d}, \end{array}$$

where *J*_*o*_ represents the inbound time spent by passengers at the starting station on the route and *K*_*d*_ represents the exit time spent by passengers at the end station on the route. *δ*_*ij*,*k*_^*w*^ and *ϕ*_*i*,*lm*_^*w*,*k*^ are the relationship between the unit interval [*i*, *j*], the transfer station *i*, and the traffic travel volume in the subway network to the path *k* between *W*.

Based on the idea of importance contribution matrix, this paper combines the structural holes and centrality features of the network to identify key sites. The K-kernel importance is used to describe the role of nodes in the network information dissemination, and the structural hole importance index and K-kernel importance index of adjacent nodes are used as the importance contribution values to reflect the local importance.

In the hidden layer of the LSTM model, the complexity of the hidden layer is increased due to the addition of three gate structures. Three different gate structures interact inside the hidden layer in a very specific way. The first structure is simpler and is often referred to as the forget gate. The input of the gate structure is the output of the hidden layer of the previous time node and the input of the hidden layer of the current node. The activation function of the gate structure is *σ*, and the output result is *f*^(*t*)^, which is sent to the upper part to interact with the *C*^(*t*)^ of the previous hidden layer. The forget gate structure can be expressed by the following formula:

(5) $$\begin{array}{c} C_{k}^{w}=J_{o}+\sum_{i,j}T_{i,j}\delta_{ij,k}^{w}+\sum_{i}e_{i}^{l,m}\phi_{i,lm}^{w,k}+K_{d}. \end{array}$$

Among them, *σ* represents the activation function of the forget gate structure, which is the sigmoid function. *W*_*f*_, *U*_*f*_, and *b*_*f*_ represent the weight parameters and biases in the forget gate structure, respectively. The middle structure is called the input gate, and the input gate structure can be expressed as

(6) $$\begin{array}{c} i^{\left(t\right)}=\sigma \left(W_{i}*h^{\left(t-1\right)}+U_{i}*x^{\left(t\right)}+b_{i}\right),\\ \tilde{C^{\left(t\right)}}=\text{tanh}\left(W_{c}*h^{\left(t-1\right)}+U_{c}*x^{\left(t\right)}+b_{c}\right), \end{array}$$

where *σ* represents the activation function of the first part of the input gate. *W*_*i*_, *U*_*i*_, *W*_*c*_, *U*_*c*_, *b*_*i*_, and *b*_*c*_ represent the weight parameters and biases of the corresponding parts, respectively. The last gate is called the output gate. The output gate structure can be expressed as

(7) $$\begin{array}{c} o^{\left(t\right)}=\sigma \left(W_{o}*h^{\left(t-1\right)}+U_{o}*x^{\left(t\right)}+b_{o}\right),\\ h^{\left(t\right)}=o^{\left(t\right)}*\tanh\left(C^{\left(t\right)}\right), \end{array}$$

where *σ* represents the activation function of the first part of the output gate. *W*_*o*_, *U*_*o*_, and *b*_*o*_ represent the weight parameters and bias of the output gate, respectively. The normalization formula of the indicator data is as follows:

(8) $$\begin{array}{c} y=\left(y_{\max}-y_{\min}\right)*\frac{\left(x-x_{\min}\right)}{\left(x_{\max}-x_{\min}\right)}+y_{\min}. \end{array}$$

Among them, *y*_min_=−1 and *y*_max_=1.

In this paper, a method for processing input real-number data using Gaussian distribution characteristics is presented, allowing the newly constructed deep belief network to process the experimental data effectively. Because each impact index has a different physical meaning and no uniform dimensional unit, it is difficult to get a satisfactory answer directly using the impact index as an input quantity, which will slow the network's convergence. This study applies dimensional processing to the data in order to make the indexes comparable. In general, due to the large range of collected data, it is generally required that the value of input data be between [−1, 1] in order to improve training speed and effectively avoid the saturation area of the sigmoid function. The DL feature extraction process can ignore the complex coupling relationship between urban rail transit vehicle equipment systems and the complex functional relationship between feature vectors, mine the data's internal structure, and find the essential relationship between variables. Robustness refers to the system's ability to provide alternate routes in the event of accidents or targeted attacks, and it is one of the key indicators used to assess the rail transit system's quality. The system's robustness is conducive to improving the urban rail transit system's reliability, service quality, and resilience, allowing it to fully exploit rail transit's obvious advantages. The LSTM model goes through the same training and optimization process as the NN model. To iteratively update all parameters in the model, it also uses a time-based back propagation algorithm and the gradient descent method. All of the parameters are based on partial derivatives of the loss function, which is the most important point. The network is trained and tested in a circular fashion in this paper, with the reasonable error range as the final error range. Finally, all of the remaining cities serve as training samples and models, with the trained models being used to research urban rail transit network route planning.

## 4. Result Analysis and Discussion

**Figure 3:**
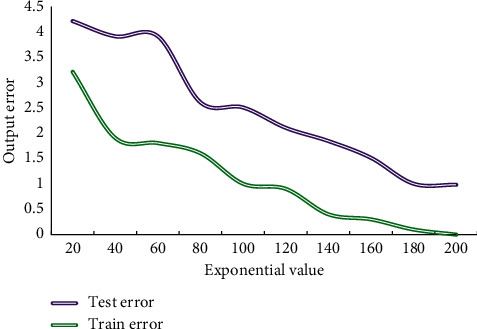
The training results of the model in this paper.

The model training platform is Windows 10, equipped with Intel 8-core CPU; memory is 32G. Python is chosen as the main programming language. TensorFlow framework in Keras not only has good usability and practicability, but also has flexibility and high efficiency, fast training speed, and flexible parameter change. It can provide corresponding operation support on multiple platforms. Therefore, this paper chooses TensorFlow in Keras as the back-end framework of DL model. After analysis and screening, 40 cities with developed urban rail transit were selected for analysis and research, and finally 12 representative cities were selected as samples for experiment. Then, the model parameters and targets are selected to be digitized. In order to get more accurate results and reasonable error range, 2 cities are selected as test samples from 12 cities, and the remaining cities are used as training samples. Run the program and record the error.  Figure 3 shows the training results of the model.

In this study, the results obtained for the established predictive model are numerical data. Therefore, this chapter selects the representative MSE (mean squared error), RMSE (root mean square error), and MAE (mean absolute error) three indicators to test the algorithm, respectively. The calculation formulas of the three indicators are as follows:

(9) $$\begin{array}{c} \text{MSE}=\frac{1}{n}{\overset{n}{\underset{k=1}{\sum }}\left(y_{k}-y_{k}^{\prime }\right)}^{2},\\ \text{RMSE}=\sqrt{\frac{1}{n}{\overset{n}{\underset{k=1}{\sum }}\left(\left|y_{k}-y_{k}^{\prime }\right|\right)}^{2}},\\ \text{MAE}=\frac{1}{n}\overset{n}{\underset{k=1}{\sum }}\left|y_{k}-y_{k}^{\prime }\right|. \end{array}$$

Among them, *n* represents the number of predicted samples, *y*_*k*_ is the actual value, and *y*_*k*_′ is the model output value. The prediction accuracy evaluation methods of the three models are suitable for DL models. By comparing the evaluation indexes among different models, we can roughly understand the performance of the models. These three indexes are used to test different algorithms, and the results are shown in Table 1.

**Figure 4:**
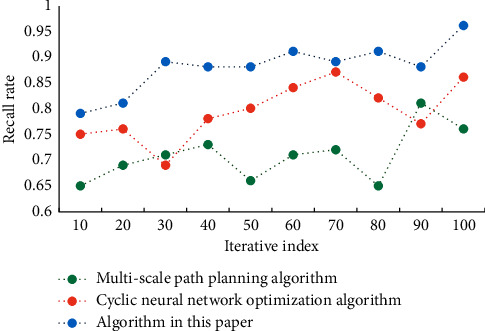
Recall results of different algorithms.

**Figure 5:**
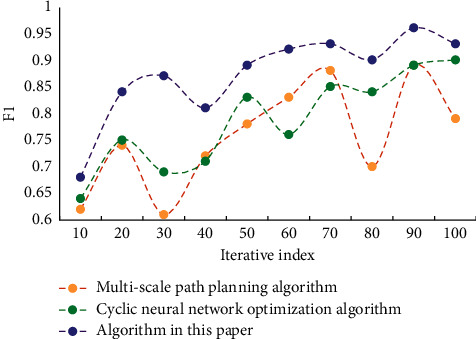
F1 value results of different algorithms.

It can be seen from the data in the table that the errors of the optimization algorithm of urban rail transit network route planning in this paper are smaller than those of the comparison algorithm, and their values are at a low level. This shows the accuracy and reliability of this algorithm.  Figure 4 shows the recall results of different algorithms.  Figure 5 shows the F1 values of different algorithms.

**Figure 6:**
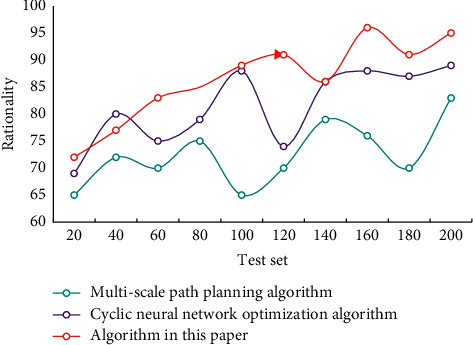
Comparison of rationality of planning with different methods.

The existing saturation of rail transit of each sample is taken as the output target value of NN, and the training sample and the inspection sample should be representative. Through training and learning, the network adapts to the nonlinear mapping relationship between the input sample and the target value, and then it is used for simulation calculation. Test the rationality of using different algorithms for urban rail transit network route planning. The rationality comparison is shown in Figure 6.

**Figure 7:**
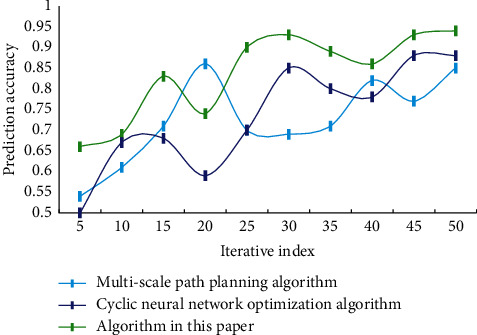
Accuracy of traffic flow prediction of different algorithms.

The prediction results of several DL models are compared one by one according to the same evaluation index, among which the traditional time series prediction method and autoregressive summation average model are added to achieve the effect of horizontal comparison. The traffic flow prediction accuracy of different algorithms is shown in Figure 7.

According to the data in Figure 7, the accuracy rate of real-time traffic flow prediction by this algorithm can reach 94.98%, which is about 9% higher than other methods. The prediction results and accuracy prove the rationality and effectiveness of the proposed prediction method. This paper simulates the actual traffic conditions.  Table 2 shows the comparison results of execution time of different algorithms.

It can be seen that under the same conditions, the execution time of the route planning optimization algorithm in this paper is short. This shows that the algorithm in this paper has higher efficiency and better performance.

Experiments in this chapter show that the accuracy rate of real-time traffic flow prediction by this algorithm can reach 94.98%, which is about 9% higher than other methods. This verifies the real time, rationality, and optimality of the proposed algorithm, which can reduce the management cost and obtain a higher input-output ratio. The algorithm proposed in this paper can achieve good results in the route planning of urban rail transit network.

## 5. Conclusions

Congestion is a major issue in today's major cities, and there are numerous solutions available. Construction of fast, large-capacity, safe, and environmentally friendly urban rail transit has almost become the default option for all major cities looking to alleviate traffic congestion. As a result, systematically analyzing and studying the functional positioning of urban rail transit network lines in the urban agglomeration regional rail transit system, integrating the regional rail transit system, and comprehensively planning and optimizing the network line layout in conjunction with the urban spatial structure and personnel travel characteristics are critical. It can effectively promote the formation of a regional high-speed comprehensive transportation system, ease people's travel, reduce traffic congestion, and lay a solid foundation for good urban traffic development. This paper proposes an optimization algorithm based on DL to address the low rationality and optimality of traditional urban rail transit network route planning. This paper describes the algorithm's design and implementation. The accuracy of this algorithm in predicting real-time traffic flow can reach 94.98 percent, which is about 9% higher than other methods, according to experimental results. This validates the proposed algorithm's real time, rationality, and optimality, lowering management costs and increasing the input-output ratio. The content of the urban rail transit mode is complex, involving a wide range of variables and numerous influencing factors. Despite the fact that this paper has made some progress in the theory and practice of urban rail transit network route planning, due to a lack of knowledge and time, there are still some issues that need to be addressed. We will expand the sample data set while appropriately deepening the model's depth, and we will determine the parameters in the model by distinguishing the samples during the week and at the weekend in the follow-up study in order to make the prediction model more applicable.

## Figures and Tables

**Table 1:** Index test results of different algorithms.

Algorithm	MSE	RMSE	MAE
Multiscale path planning algorithm	0.157	0.437	0.658
Cyclic neural network optimization algorithm	0.096	0.396	0.437
Algorithm in this paper	0.049	0.215	0.426

**Table 2:** Execution time of different algorithms.

Group	Starting point	Target point	Multiscale path planning algorithm	Cyclic neural network optimization algorithm	Algorithm in this paper
1	396	3678	894	136	81
2	579	3541	1103	94	46
3	863	2796	1039	87	51
4	827	3024	1004	98	58
5	978	4169	936	86	29

## Data Availability

The data used to support the findings of this study are available from the corresponding author upon request.
